# Predictors of posttraumatic growth among conflict-related sexual violence survivors from Bosnia and Herzegovina

**DOI:** 10.1186/s13031-019-0201-5

**Published:** 2019-06-04

**Authors:** Kimberley Anderson, Amra Delić, Ivan Komproe, Esmina Avdibegović, Elisa van Ee, Heide Glaesmer

**Affiliations:** 1 Psychotramacentrum Zuid Nederland, Reinier van Arkel Groep, Amsterdam, The Netherlands; 2 Department for Medical Psychology & Medical Sociology, University of Leipzig, Philipp-Rosenthal-Str. 55, 04103 Leipzig, Germany; 3 Department of Psychosomatic Medicine and Psychotherapy, HELIOS Hanse Hospital Stralsund, University of Greifswald, Greifswald, Germany; 4 HealthNet TPO, Amsterdam, The Netherlands; 5 University of Tuzla, Department for Psychiatry, Tuzla, Bosnia and Herzegovina; 6 Behavioural Science Institute, Radboud University, Nijmegen, the Netherlands; 7 Faculty of Social and Behavioural Sciences, University of Utrecht, Utrecht, The Netherlands

**Keywords:** Sexual violence, Rape, Conflict, War, Bosnia and Herzegovina, Posttraumatic growth, PTGI, Optimism, Coping

## Abstract

**Background:**

Conflict-related sexual violence (CRSV) was committed on a large scale against women across Bosnia and Herzegovina (BiH) during the 1990’s war, and research has shown both negative and positive psychosocial outcomes following such acts of interpersonal violence. We aim to determine the capacity for posttraumatic growth (PTG) among a population of women who experienced CRSV, and to what extent it is impacted by factors such as coping and optimism.

**Methods:**

This study sought to examine the relationship between PTG (posttraumatic growth inventory), symptoms of posttraumatic stress disorder (PTSD; Harvard Trauma Questionnaire) and dispositional factors such as coping (COPE) and optimism (Life-Orientation Test-Revised) in a sample of *n =* 104 women. We first conducted bivariate correlations and then hierarchical linear regression analyses, and hypothesized that approach coping strategies and optimism will act to enhance PTG.

**Results:**

Findings showed that the average total score for PTG in this study was 58.94 (SD = 23.01), and current PTSD symptomatology above a threshold of > 2.5 was detected in 92.3% (*n* = 96) participants (mean score 3.18, SD = .45). Bivariate correlations showed that higher levels of PTG were associated with greater optimism, greater approach coping strategies positive reinterpretation and planning, and lower avoidance strategies behavioural disengagement and substance use. When entered into a regression model, only positive reinterpretation and behavioural disengagement remained, the *R*-square of the total set of predictors was 0.16, thus explaining 16% of PTG total score.

**Conclusion:**

Two types of coping (namely capacity of both greater positive reinterpretation and lower behavioural disengagement) most strongly predicted growth after trauma in this sample of CRSV survivors from BiH. These dimensions of coping confirm the role of coping strategies in the development of PTG. Further research would be useful in corroborating these findings in other post-conflict settings, and delving further into the possibility of a dual mechanism of growth and distress after CRSV.

**Electronic supplementary material:**

The online version of this article (10.1186/s13031-019-0201-5) contains supplementary material, which is available to authorized users.

## Introduction

In late 1992, shortly after Bosnia and Herzegovina (BiH) declared independence from the former state of Yugoslavia, the first bombs were dropped on its capital city, Sarajevo. For the next three years, conflict tore its way through the country, impacting the lives of virtually all its people [1, 2]. Sexual violence – including kidnap, sexual slavery, sexual exploitation and human trafficking – was a prominent feature of this conflict [3]; whereby women are believed to have been targeted by civilians; locally-stationed and foreign military forces [4–7] and United Nations Peacekeepers [8]. In a study of 51 female rape survivors conducted in BiH more than 20 years after the start of the war [9], results showed 57% still suffered from posttraumatic stress disorder (PTSD), 76% reported disrupted sleep and 40% expressed frequent thoughts about death and dying. Such results indicate the long-term, debilitating impact of conflict-related sexual violence (CRSV) on survivors in BiH, yet, the power of these women should be given equal address. In the words of one survivor from the same study: “we are not ‘those poor women’, we are strong, active and courageous”. A shift to focusing on salutary outcomes, for example posttraumatic growth (PTG) and assets central to promoting health and supportive environments, could be fundamental in achieving lasting, positive health outcomes after negative life events [10]. A pertinent issue however, is if/how salutogenic (e.g. PTG) and pathogenic (e.g. PTSD) outcomes are related, in particular whether the presence of both in the aftermath of CRSV is possible.

Posttraumatic growth (PTG) has been defined over recent decades as a beneficial change in cognitive and emotional capacities beyond previous levels of adaptation, psychological functioning, or life awareness [11], and should not be used interchangeably with positive psychological and emotional attributes, such as resilience or thriving [12, 13]. Posttraumatic growth instead refers to permanent change [11] and what it means to live optimally in the wake of trauma. It is argued that the process of PTG is triggered by the onset of a traumatic stressor, severe enough to challenge previously held beliefs; which is then followed by a pivotal second phase of reflective processing [14, 15]. Growth is thought to occur during this second phase as individuals derive meaning from their traumatic experiences, facilitating the cognitive restructuring of their beliefs and assumptions of the world. Solomon & Dekel [16] hypothesize that the suffering a person manoeuvres during this second phase of restructuring is most important to subsequent outcomes. They hypothesize that following trauma, PTG can arise regardless of the severity of the event and symptoms of PTSD, which likely means that positive and negative reactions emerge as related, but separate outcomes.

Several key factors may enhance or impede the development of PTG and PTSD, but there currently exists a relatively limited evidence base that focuses on growth following interpersonal events such as CRSV. We will focus on two prominent dispositional aspects – coping and optimism – in the following, with the hope that by understanding their impact on other similar populations, we can determine their importance in supporting CRSV survivors towards recovery. A study by Ai and colleagues [17] assessed Bosnian refugees now resettled in the USA, on measures of coping and hope. Results showed that on the whole, a higher number of war-related traumatic events was associated with negative patterns of coping, potentially leading to poorer overall health. Using the widely accepted categories of coping defined by Lazarus [18], Littleton [19] similarly posits that the use of avoidance coping strategies (that allow a person to avert the source of the stress) are commonly associated with greater distress and negative outcomes in the aftermath of rape, the author states that the tendency to suppress thoughts about the stressor may in fact lead to over-attention to the event. More recently however, positive relationships between avoidance strategies and growth have been observed, which are believed to mitigate acute emotional distress following trauma. London, Mercer, & Lilly [20] explain that this interaction which causes traumatic events to evoke adaptive cognitive efforts and lead to greater experiences of PTG, may actually be overcompensating, resulting in protective coping mechanisms of a deceptive nature. Thus, we may see higher self-reported PTG scores as a means of self-preservation. For those who successfully manoeuvre trauma, the true transformative component of PTG is assumed to develop over time, while this illusory component diminishes [21]. Avoidance is also a core component of PTSD, which may contribute to explaining a greater severity of PTSD symptoms at the same time as PTG [22]. In other populations, however, research among patients with a history of coronary episodes suggests that avoidance coping strategies can lead to more positive adaptations in the short term, but have no or even negative effects in the long term [23].

Another dispositional factor is how individuals anticipate stressful situations and other life adversities; whether or not they tend to expect positive outcomes. It is widely understood that optimistically inclined people can derive a sense of benefit from adversity, to a greater extent than pessimistic people, and that dispositional optimism is understood to be generalized and stable over time [24, 25]. But other factors can influence these patterns, and increase the risk for onset and relapse of psychopathology both directly and indirectly [26]. According to Solberg Nes & Segerstrom [27], optimists tend to be flexible in their approach to coping; more likely using a range of strategies to ensure a greater sense of perceived control over stressors and traumatic events. However, if a person remains wholly optimistic, while denying the existence of negative outcomes, the self-perception of PTG may turn into one of avoidance, resulting in negative effects on adjustment [21].

Much existing literature does not reflect the specific complexities of sexual violence in the context of war and the possibility for growth, while remaining in a country during post-conflict times. These can include the negative appraisal of emotions and symptoms, perceived negative responses from others, and the confrontation of shame and stigma [28, 29]. Part of the challenge in resolving distress in the aftermath of CRSV, is that gaining a sense of empowerment is often achieved through taking action [22], which avoidance coping mechanisms may prevent from happening. We believe that understanding the capacity for growth in survivors of CRSV is important and necessitated at a time when there is comparably less attention drawn to the positive outcomes of trauma. Exploring the possibility for PTG to occur in the aftermath of conflict and investigating the role of dispositional factors (such as coping and optimism) is an important avenue within post-conflict research. Yet, a greater understanding of these will be relevant and beneficial to mental health professionals across multiple settings, in being better able to deliver improved, survivor-centred care.

This may include using a person’s existing emotional, social and spiritual resources, understanding how these factors might buffer against poor mental well-being among survivors of CRSV, and how these can be applied in post-conflict settings and beyond.

On the basis of the literature outlined above, the objectives of this study were therefore to: 1) determine the extent of PTSD and PTG among a sample of female CRSV survivors from BiH, and 2) examine the relevance of coping and optimism in this relationship. The core assumption is that approach coping strategies and optimism will promote PTG for the sample of CRSV survivors in the present study, and avoidance coping styles and high degree of pessimism will act as a risk factor in the distress-growth relationship.

## Methodology

### Design and setting

The data presented here are part of a cross-sectional study on the quality of life and long-term psychological consequences in women with experience of CRSV during the conflict in BiH. Data were collected from December 2012 to December 2014 in seven regions across the country. The study was approved by the Human Research Ethics Board of the Medical Faculty of the University of Tuzla, Bosnia and Herzegovina.

### Participants

Female survivors of CRSV in this study were recruited through the Bosnian non-governmental, non-profit *Association for Women Victims of War*, who hold a database of sexual violence survivors (SSV). Participants were eligible for inclusion if they were residents of BiH during and after war, and between 30 and 65 years old at the time of study. The President of the Association for Women Victims of War contacted sexual violence survivors (SSV) to provide them with information about the research project and to invite them to voluntarily participate in the study.

For those who assented, the interview was conducted by author AD, who is trained as a psychiatrist and researcher, in two or more sessions, with breaks upon request. As some of the questions were sensitive, referral information for psychological counselling and support was provided. On the basis of a study screening measure of cognitive functioning (Mini Mental Status Examination, MMSE; Folstein, Folstein, & McHugh [30]) women were excluded with scores < 23 or those with a psychiatric history prior to the war.

Interviews were carried out with a total of 110 SSV; of which three later withdrew their consent, two were excluded due to MMSE < 23, and one participant was excluded due to a large amount of missing data. The present study therefore comprised a sample of 104 female survivors of CRSV, with an average age of 48.8 years (SD = 8.7). The majority of women were of Bosniak ethnicity (90.6%, *n* = 94.2) with the rest a mixture of Bosnian Croat (4.4%, *n =* 4.6) and Bosnian Serb (5%, *n =* 5.2). Over half of participants were married (56.7%, *n* = 59), exactly half (50%, *n* = 52) of women had attended school up to secondary level, and just over two thirds (68.7%, *n* = 71) were not currently employed outside of the home.

### Instruments

*Socio-demographic questionnaire:* A self-developed questionnaire was designed by author AD to gather sociodemographic data (including ethnic affiliation, age, marital status, education, employment status, the context of CRSV, and any participation in psychosocial support programs).

*Posttraumatic Growth Inventory (PTGI; Tedeschi & Calhoun* [31]*):* The PTGI is a 21-item self-report scale for assessing psychological growth following a traumatic event, in this case framed in the context of participants’ experiences of CRSV. The PTGI includes five subscales: new possibilities (e.g., “established a new path for my life”), relating to others (e.g., “a sense of closeness with others”), personal strength (e.g., “knowing I can handle difficulties”), spiritual change (e.g., “I have a stronger religious faith”), and appreciation for life (“I appreciate each day”). Items range from 1 (“I did not experience this change as a result of my crisis”) to 6 (“I experienced this change to a very great degree as a result of my crisis”) and total scores on the PTGI range from 1 to 126, with higher scores reflecting greater perceived growth. In the present study, Cronbach’s alpha for the PTGI total score was 0.96 and ranged from 0.66 to 0.90 for subscale scores.

*Harvard Trauma Questionnaire (HTQ; Mollica, Caspi-Yavin, Bollini, & Truong* [32]*):* A version for Bosnia and Herzegovina [33] was used to explore experiences of traumatic events and assess the presence of PTSD symptomatology. It consists of four parts, though only the first and fourth parts were used in this study. The first part is a list of possible traumatic events that civilians could potentially have been exposed to during the war, for which there is a yes/no response format. Events include: material deprivation, conditions relating to war, bodily injury, forced confinement and coercion, being forced to harm others, disappearance, death or injury to loved ones, and witnessing violence to others. The fourth part contains 40 statements about possible psychosocial difficulties caused by trauma. The first 16 statements are derived from DSM-IV criteria for PTSD and inquire about the symptoms of re-experiencing the traumatic event, avoidance, and hyperarousal. The scale for each response in these sections are rated from 1 “not at all” to 4 “very strongly”, whereby a mean item score of 2.5 or above from the 16 items is considered an indicator of symptoms of PTSD. In this study the HTQ demonstrated very high reliability and internal consistency, with Cronbach’s *α* = 0.96 for the PTSD symptom scale.

*Life-Orientation Test – Revised (LOT-R; Scheier, Carver, & Bridges* [34]*):* The LOT-R is a 10-item self-report measure of dispositional optimism and pessimism, and given its validation in Serbia [35], it was deemed applicable for use in the Bosnian population. It focuses on positive and negative expectations for the future regardless of the means by which outcomes occur. In the 1994 revised version, the items more closely focused on expectancies of the future, which is the core of the conceptual trait of dispositional optimism. Three items in the scale capture optimism (Cronbach’s α = .70), three capture pessimism (Cronbach’s α = .74) and the four remaining items are fillers and are not scored. Scores were calculated in its original unidimensional format. Respondents rate each item on a scale from 0 “strongly disagree” to 4 “strongly agree” (*α* = .74). A total score is calculated by adding the inverted pessimism score to the optimism score. Cronbach’s *α* for the total score is .73.

*Coping Orientations to Problems Experienced Scale (COPE; The Bosnian version used in this study is an adaption of the validated Croatian COPE scale, Hudek-Knežević, Kardum, & Vukmirović* [36]*)*: The Croatian COPE is a 71-item scale to assess how people respond when they are confronted with difficult or stressful situations, whereby respondents rate each statement on a scale from 0 “never” to 4 “I always do this”. It produces 15 subscales that mirror the original COPE-60 [37], which include problem-focused, emotion-focused and disengagement dimensions, and reflect the activities from a particular coping domain. In the validated Croatian version, ‘substance use’ referred exclusively to alcohol, in the Bosnian translation, this referred only to the use of sedatives. In the present study, the internal consistency for 12 of the 15 subscales ranged from .57 to .89. For three, Cronbach’s alpha was .50 or below (restraint .50, denial .44 and suppression of competing activities .44), though this scale has been used successfully in existing research (see Antičević, Kardum, & Britvić [38]).

For the measures without validated Bosnian scales, a process of translation and back translation was conducted among the research team, students and co-workers at the University of Tuzla, Department for Psychiatry.

### Data analyses

As an exploratory study, we were primarily interested in the presence of PTG and its predictors, so as to create a basis for further exploration. In order to do this, the associations of study criterion variables (PTGI and the five related subscales) were estimated with various study predictor variables (severity of PTSD, optimism, coping) in a two-step process. First, a correlation matrix was calculated to explore relationships between predictor and criterion variables. Second, the predictor variables that significantly correlated with the study outcome variable or one of its subscales were entered as clusters in separate steps in multivariate linear regression analyses. For the outcome variable PTG and its subscales, we estimated adjusted *R* squared *(ΔR*^*2*^*)* and beta *(β)* values for each (set of) predictor(s). *ΔR*^*2*^ shows the proportion of explained variance of the outcome variable by all analysed predictors, and *β* shows the relative contribution of a predictor in explained variance of the outcome variable. For all tests used in this study, differences were considered significant when *p* = < .05. There were few missing data within the sample, but where missing values existed we calculated the series mean. We factored in the total number of scale items when doing this, and excluded scores where more than 70% of values were missing. To prevent multicollinearity, continuous variables were standardized (z-scores). All analyses were performed with SPSS version 25 (IBM, 2018).

## Results

### Circumstances of CRSV

Regarding the circumstances of rape, 45.2% (*n* = 47) of women indicated that they were raped three or more times during the conflict, with almost a third (29.8%, *n* = 30) having been raped by three or more perpetrators. The vast majority of women (76%, *n* = 79) did not know their perpetrators. Women were on average 29.56 (SD = 8.9) years old (range 12–48) when their first experience of rape took place. Fourteen women (13.5%) expressed that they became pregnant as a result of being raped, of whom 10 (9.6%) had this pregnancy terminated.

### Severity of PTG, PTSD and psychosocial support

Table 1 gives an overview of total scores of predictor and criterion variables. The average score for PTG in this sample was 58.98 (SD = 23.01) out of a possible score of 126. Participants scored highest on factors 1 and 2 (‘relating to others’ and ‘new possibilities’). Using the HTQ as a screener for PTSD, the average item score for participants in this sample was 3.18 (SD = .45). Given the defined threshold of scores > 2.5 as an indication of PTSD symptomatology, 96 participants (92.3%) in this sample screened positive. The average number of traumatic events experienced by this sample across the lifetime was 25.52 (SD 5.8) events (range 7–39). Despite this, less than a third of women in this study (*n* = 29, 28%) have sought psychosocial support since the end of the war in BiH, with one person reportedly accessing specialist care for CRSV survivors. The average period of silence of their experience of rape was 10.52 (SD = 5.9) years.

**Table 1:** Socio-demographics and total scores for study predictor and criterion variables

	Mean	SD	
Age	48.85	8.72	
	n	(%)	
Ethnicity			
Bosnian	94	90.4	
Croatian	5	4.8	
Serbian	5	4.8	
Marital status			
Married	59	56.7	
Single	12	11.5	
Widow	22	21.1	
Divorced	11	10.6	
Education			
No schooling	14	13.5	
Primary education	32	30.8	
Secondary education	52	50.0	
Higher education	6	5.8	
Work			
Employed	16	15.4	
Unemployed	71	68.3	
Retired	17	16.3	
	**Mean**	**SD**	**Range**
PTGI Total score	58.94	23.01	0–126
PTGI Subscales			
PTGI Factor 1: Relating to others	19.12	8.03	0–35
PTGI Factor 2: New Possibilities	17.38	6.17	0–25
PTGI Factor 3: Personal Strength	11.38	4.95	0–20
PTGI Factor 4: Spiritual Change	6.82	2.52	0–10
PTGI Factor 5: Appreciation of Life	9.04	3.89	0–15
HTQ			
PTSD	3.18	.45	Cutoff > 2.5
No. events	25.2	5.8	
LOT-R	11.36	3.45	0–24
COPE Subscales			
Positive reinterpretation	9.1	2.86	0–16
Active coping	10.26	2.59	0–16
Planning	13.7	3.43	0–20
Acceptance	10.66	2.6	0–16
Restraint	9.23^a^	2.54	0–16
Suppression of competing activities	12.10^a^	2.78	0–20
Denial	7.63^a^	3.33	0–16
Behavioural disengagement	8.65	3.74	0–20
Mental disengagement	14.99	4.06	0–24
Use of emotional social support	8.07	3.66	0–16
Use of instrumental social support	7.93	3.35	0–16
Venting of emotions	7.38	2.57	0–12
Humour	3.6	2.74	0–12
Substance use	13.63	4.73	0–20
Religion	13.52	2.5	0–16

*n =* 104

*PTGI* Posttraumatic growth inventory (total score 1–126)

*LOT-R* Life-Orientation Test Revised (score range 0–24)

*COPE (Bosnian)* Coping Orientations to Problems Experienced Scale

*HTQ (Bosnian)* PTSD mean score items 1–16 (2.5 screening threshold), No. events across lifetime

^a^Alpha below .50

### Associations between variables

By calculating bivariate correlations, the following variables exhibited significant associations with PTGI total score (*p* < .05): optimism (*r* [103] = 0.285), and coping subscales: positive reinterpretation (*r* [103] = 0.336), planning (*r* [103] = 0.282), behavioural disengagement (*r* [103] = − 0.249) and substance use (*r* [103] = − 0.211,). Complete bivariate correlations of criterion variable PTGI total score and its subscales, and predictors, can be found in the Additional file 1.

### Predicting PTG

Predictor variables that were significantly correlated (*p* = < .05) with the study outcome variable (PTGI total score) or one of its subscales in step 1 of the analysis (see Table 2), were hierarchically entered as clusters in separate multivariate linear regression analyses as step 2 of the analysis. Given the high prevalence of PTSD symptomatology across the sample, we controlled for this in the analysis by entering it in each regression as cluster 1. Optimism and coping subscales were entered forward in cluster 2.

**Table 2:** Predictors of PTG (PTGI Total Score and subscales)

Predictors	*β*	*SE*	*t*	*p*	*R* ^*2*^	*ΔR* ^*2*^
PTGI Total Score						
Step 1					.020	0.01
HTQ	0.141	.098	1.434	0.155		
Step 2					.128**	.110
HTQ	0.12	.093	1.288	2.01		
COPE Positive reinterpretation	.329	.093	3.535	.001**		
Step 3					.182**	.157
HTQ	0.183	.094	1.949	.054		
COPE Positive reinterpretation	.282	.092	3.047	.003**		
COPE Behavioural disengagement	−.245*	.095	−2.569	.012*		
PTGI Factor 1: Relating to others						
Step 1					.009	−.001
HTQ	.094	.099	.949	.345		
Step 2					.105**	.087
HTQ	.083	.094	.878	.382		
LOT-R	.310	.094	3.291	.001**		
Step 3					.142**	.116
HTQ	.072	.093	.777	.439		
LOT-R	.253	.097	2.619	.010*		
COPE Positive reinterpretation	.201	.097	2.072	.041*		
PTGI Factor 2: New possibilities						
Step 1					.018	.009
HTQ	.136	.098	1.383	.170		
Step 2					.115**	.098
HTQ	.116	.094	1.238	.219		
COPE Positive reinterpretation	.312	.094	3.329	.001**		
Step 3					.163**	.138
HTQ	.112	.092	1.224	.224		
COPE Positive reinterpretation	.247	.096	2.587	.011*		
LOT-R	.229	.095	2.396	.018*		
PTGI Factor 3: Personal strength						
Step 1					.026	.017
HTQ	.162	.098	1.654	.101		
Step 2					.153*	.136
HTQ	.139	.092	1.518	.132		
COPE Positive reinterpretation	.357	.092	3.889	.000**		
Step 3					.226*	.203
HTQ	.186	.089	2.078	.040*		
COPE Positive reinterpretation	.336	.088	3.805	.000**		
COPE Substance use	- .276	.089	−3.081	.003**		
Step 4					.267*	.238
HTQ	.233	.090	2.599	.011*		
COPE Positive reinterpretation	.298	.088	3.383	.001**		
COPE Substance use	−.221	.091	−2.438	.017*		
COPE Behavioural disengagement	−.220	.094	−2.352	.021*		
PTGI Factor 4: Spiritual change						
Step 1					.026	.017
HTQ	.162	.098	1.654	.101		
Step 2					.153**	.136
HTQ	.139	.092	1.518	.132		
COPE Positive reinterpretation	.357	.092	3.889	.000**		
Step 3					.226**	.203
HTQ	.186	.089	2.078	.040*		
COPE Positive reinterpretation	.336	.088	3.805	.000**		
COPE Substance use	- .276	.089	−3.081	.003**		
Step 4					.267**	.238
HTQ	.233	.090	2.599	.011*		
COPE Positive reinterpretation	.298	.088	3.383	.001**		
COPE Substance use	−.221	.091	−2.438	.017*		
COPE Behavioural disengagement	−.220	.094	−2.352	.021*		
PTGI Factor 5: Appreciation of life						
Step 1					.016	.007
HTQ	.128	.098	1.303	.195		
Step 2					.088*	.070
HTQ	.196	.098	1.998	.048*		
COPE Behavioural disengagement	−.276	.098	−2.818	.006**		
Step 3					.124**	.097
HTQ	.174	.097	1.791	.076		
COPE Behavioural disengagement	−.237	.099	−2.401	.018*		
COPE Positive reinterpretation	.193	.096	2.013	.047*		

*p < .05 **p < .01

*PTGI* Posttraumatic growth inventory

*LOT-R* Life-Orientation Test Revised - Optimism-pessimism

*COPE (Bosnian)* Coping Orientations to Problems Experienced Scale

*HTQ (Bosnian)* PTSD mean score items 1–16

Overall, combinations of optimism and four types of coping significantly predicted PTGI total score and each of the five subscales. The results of the regression analyses indicate that two predictors explained the largest proportion of variance: *ΔR*^*2*^ .157 of PTGI total score, with standardized regression coefficients for positive reinterpretation *β* .282 (*t* = 3.05, *p* < .01) and behavioural disengagement *β* − .245 (*t* = − 2.57, *p* < .05). Table 2 summarizes the results of the multivariate regression analysis for PTGI total scores and all five subscales.

## Discussion

This study sought to explore the extent of PTG among a sample of female CRSV survivors from Bosnia and Herzegovina, and to further understand positive and negative outcomes of trauma, by examining the relationship between PTG, PTSD symptomatology and dispositional factors such as coping and optimism. To the best of our knowledge, this is the first study to assess these combined measures within the context of CRSV, and the first within BiH. The main findings in relation to our first objective show the overall score for PTG was 58.94. Comparative to findings documented in existing literature, this score sits in the mid to high range among samples of sexual and physical assault, and rape survivors (see a review by Elderton, Berry, & Chan [39], for scores ranging from 32.76 to 68.08). With regard to populations of refugees and displaced persons among the Bosnian population who were exposed to war-related trauma, this is comparatively high (see Powell et al. [40] for an average score of 44.10) but low when compared to students in the general population (average score 83.16, see Tedeschi & Calhoun [31]). Upon closer investigation, participant scores indicate that PTGI factor 1: relating to others (i.e. gaining an appreciation of closeness with others, making effort in relationships) is the domain in which they perceived the most growth, with the second highest associated with factor 2: new possibilities (i.e. planning for the future, seeking new opportunities). The lowest mean scores in this sample were associated with factor 4: spiritual change.

Scores of dispositional optimism in this study were almost 50% lower than in a sample of Bosnian refugees resettled in the Netherlands [1]. This finding could potentially be a reflection of optimism which is influenced by context, as a central component to growth following trauma. The study by Mooren & Kleber [1] assessed women who had the prosperity of a future in a different country without war. Bosnian survivors of CRSV who remained in the country post-war were often displaced to regions where conflict was ongoing (even after peace was officially declared). This is linked to the widespread narrative that victims of war have been largely neglected by the Bosnian government and the international community, with decades-long prosecution of perpetrators [5]. These findings are consistent with other studies of mental health outcomes of individuals from Balkan countries who remained after the war compared to those who fled the region as refugees. For example, results from Priebe and colleagues [41] found there to be less improvement in PTSD symptomatology among those who remained in post-conflict settings, and posit that complex presentations of war-related traumatic stress, coupled with higher levels of other risk factors, such as post-war socioeconomic hardship and societal instability, could delay recovery and hamper optimism. Nonetheless, it must also not be discounted that successful cultural assimilation into new environments can be compromised by negative experiences in local communities, as well as displacement and migration [42], and thus a true comparison of scores of optimism between refugees and persons who did not flee, women who experienced CRSV and those who did not, and across studies of the past twenty years, remains tenuous. Equally, without cueing participants to focus specifically on CRSV experiences when reporting optimism and other outcomes, symptomatology related to more recent experiences of ongoing hardships may dominate their narrative.

The analyses with regard to our second objective, sought to further examine the relationship between PTG and associated predictors. In our sample of CRSV survivors, results showed that a higher level of PTG positively correlated with a greater degree of dispositional optimism, positive reinterpretation and planning as approach coping strategies, and negatively correlated with behavioural disengagement and substance use as avoidance coping strategies. When entered into a model to determine the power of these variables in predicting PTG overall, only positive reinterpretation and behavioural disengagement remained, and collectively accounted for 16% of growth following CRSV in this sample. The same combination of predictors of PTG (i.e. optimism, positive reinterpretation, behavioural disengagement and substance use) was consistently strongest across all PTGI subscales. These could be said to be proxy indicators of pathogenic/salutary outcomes: optimism and positive reinterpretation as buffers against, and substance use and behavioural disengagement as risk factors for, poor growth.

The role of PTSD in this sample is one of particular interest. Almost the entire sample met threshold for symptomatology, a finding which is comparable to evidence in other samples of sexual violence survivors [43]. This may be as a result of the complexities of sexual violence in the context of war, including negative appraisal of emotions and symptoms, perceived negative responses from others, and shame and stigma [28, 29] associated with this type of violence. Despite this, PTSD symptomatology (measured by HTQ mean score items 1–16) did not significantly correlate with PTG overall, nor did it remain as primary predictor when entered into a regression model for PTGI total score. This corroborates a review of research by Elderton et al. [39], who identified multiple non-significant relationships between PTG and PTSD across studies of female physical and sexual assault victims. The authors posit that growth gains are possible while still experiencing substantial distress. It is likely that distress actually forces some individuals to create meaning from their experience, and thus balance out feelings of loss or suffering. This phase of reflective processing that takes place post-trauma – whereby an individual navigates a period of cognitive restructuring (Cann et al. [14]) – may well play a pivotal role in the amount of growth that can be achieved. In particular, hyper-arousal – often regarded as the core symptom cluster of PTSD that directs subsequent trauma outcomes – may cause an individual to re-evaluate their life and relationships in the process of growth, balancing interaction with the world with withdrawing socially and engaging in avoidant behaviours [15]. This argument would also support the notion that irrespective of the severity of the trauma, pathogenic and salutary outcomes are both possible (Solomon & Dekel [16]; Grubaugh & Resick [43]); that PTSD and PTG likely therefore co-exist in a dual mechanism but are not part of the same dimension.

Questions remain though, as to what other factors dictate how well a person navigates the phase of cognitive restructuring. There is potential for pre-traumatic levels of individual resources to be indicative of capacity for growth, i.e. someone with greater personal strength may experience both more severe PTSD but also higher levels of PTG when compared against someone else who experienced the same trauma. This line of reasoning follows the temporal stability and generalized, consistent nature of optimism and coping as dispositional traits [24]; perhaps able to change slowly across the lifespan, but unchanging on the whole. And thus, although this is a cross-sectional study, we can hypothesise they are present pre-trauma, and prevail in the instance of a traumatic event, which allows us to posit a longitudinal understanding. This is a notion supported elsewhere that growth is an interactive function of pre-event resources, event appraisals, and coping strategies (Solomon & Dekel [16]). This phase could equally be altered by access to, and uptake of, psychosocial services. Only one woman in this study utilised specialist support for survivors of rape; although approximately a third of women have had access to generalised psychiatric care at some point. This result highlights a gap in service provision with regard to poor mental health post-conflict, particularly for rape survivors. It further highlights the need for a sustained interaction between psychiatric and social care facilities given the range of war-related experiences, post-war instability and chronic mental ill health. This corresponds with the premise of Mittelmark & Bauer [10] that the emphasis must remain on achieving lasting, positive health outcomes – particularly in the aftermath of traumatic events. Nonetheless, further investigation is needed to understand the development of PTG, as well as to discern the ways in which post-trauma experiences (e.g. access to care, negative social appraisal) may influence its development.

### Limitations

Although the study has some major strengths, several limitations must be considered. Firstly, self-report measures were used as the primary source of data collection, and are therefore based on perceived capacities and not on objective indicators. This is especially relevant for PTSD, for which we used a screener of symptoms but requires a subsequent clinical interview to confirm an individual diagnosis. Second, in the validated Croatian version of the COPE, substance use refers exclusively to alcohol, whereas in the Bosnian adaption this refers to the use of sedatives, unlike the original scale. Such discrepancies in translation might be reflected in the low internal consistency of multiple subscales (three with α below .50), and is cautionary in terms of how consistent these results are likely to be. Low internal consistency might also represent a lack of cross-cultural validity of the different defined coping domains, that is, the set of coping activities clustered as a subscale in the original instrument might not be contextually valid among this specific population of Bosnian women who experienced interpersonal violence in a politicised environment. Regardless, the ways in which an individual copes is by no means a guarantee that these activities are beneficial to another. On the basis of this, we consider scores on the COPE subscales as indicators of application: a low score reflects little use of the specific collection of coping styles, whereas a high score refers to a greater use of the specific collection of coping styles. Thus, in our analyses, significant relationships between coping strategies and PTG refer to the finding that higher intensity of specific coping styles are associated with higher score on PTG.

Third, given the hard-to-reach population at the focus of investigation it may be the case that this sample does not truly represent the population under study. In addition, having participants selected from a pool of war victims may imply a selection bias and explain the high rates of trauma seen in this sample. Results must be interpreted with these points in mind. It is also questionable that participants were contacted by the President of the Association for Women Victims of War, and may have felt a certain pressure to take part that would not have been felt with an external researcher. Fourth, PTSD and PTG symptoms could be related to any of the several traumatic events experienced by the sample, since it is not possible to cue measure solely to the experiences of CRSV, and not any events post-trauma. While the results can explain outcomes for this population, it cannot discount the impact of other traumatic events over the life course. Finally, studies of cross-sectional design limit the extent to which findings can be interpreted as causal evidence, or adequately reflect the course of PTG (Maercker & Zoellner [21]) or severity of PTSD symptomatology. However, these do represent temporal stability, and future longitudinal research would add weight to these ideas.

### Implications

With the scores of PTG in this sample in the mid- to high-range when compared to other populations of sexual violence victims, there is assurance that growth after CRSV is possible, even among individuals who experience political instability and economic disparity for many years in post-conflict periods. It is especially promising that even though the sample in this study is a highly burdened population, PTG remains rather high when compared to the general population. These findings should provide a basis in which to explore PTG within other similar populations and add weight to the premise of harnessing strength and new levels of life awareness and adaption; a shift to promoting positive health outcomes and supportive environments. Clinical practice and research fields, as well humanitarian professionals working in conflict-affected communities, can equally address these issues. Mental health professionals may orient their psychosocial support to harness the potential for growth, particularly if utilising the different components of PTG as outlined by Tedeschi & Calhoun [31]. For instance, this could include involving family members or connecting survivors with people who have similar histories; promoting new opportunities for social interaction or personal/professional development; psycho-education to enhance and build coping strategies; or by being attuned to survivors’ faith or religious beliefs. These findings also complement the survivor-centred approach [44], implemented by humanitarian professionals, that seeks to build individual and community resources in the aftermath of CRSV, particularly when moving from emergency to recovery phases. Practitioners may use coping strategies as identifiers as to who may be at greater risk of poor outcomes long-term, and incorporate cognitive techniques that enable survivors to reframe their trauma into a positive life experience. Researchers can explore further the particularities of coping and PTG for survivors of CRSV (in the Bosnian population), in the context of post-war instability, at different stages of recovery and if/how this has changed their perspective.

## Conclusion

Several main findings have been identified from this study, which have implications across both clinical practice and research. Namely, that optimism, approach coping strategies positive reinterpretation and planning, and avoidance strategies behavioural disengagement and substance use are most strongly associated with PTG in this sample. Upon further examination, positive reinterpretation of events and behavioural disengagement from goal-oriented activities appear to be the two main components predicting PTG, explaining approximately 16% of the variance. Given that these results likely act as proxy indicators of pathogenic and salutary outcomes, as both protective and risk factors respectively, addressing their power in a therapeutic capacity could promote growth gains overall. These findings reiterate the idea that growth does not equate to simply a decrease in distress or an increase in well-being, but is likely a more complex, dual mechanism. Future research can explore other impinging factors that contribute to PTG among populations of CRSV survivors.

## Additional file

Additional file 1: **Table S2.** Bivariate correlations of study predictor and criterion variables (DOCX 35 kb)

## Abbreviations

BiH: Bosnia and Herzegovina
COPE: Coping Orientations to Problems Experienced Scale
CRSV: Conflict-related sexual violence
HTQ: Harvard Trauma Questionnaire
LOT-R: Life-Orientation Test Revised
MMSE: Mini mental state exam
PTG: Posttraumatic growth
PTGI: Posttraumatic Growth Inventory
PTSD: Posttraumatic stress disorder
SSV: Survivor of sexual violence
