# Supplementary material for: Predictors of posttraumatic growth among conflict-related sexual violence survivors from Bosnia and Herzegovina
Source: Confl Health. 2019 Jun 4;13:23. doi: 10.1186/s13031-019-0201-5 (PMC6549258; doi:10.1186/s13031-019-0201-5)
Supplement: Supplementary file 1 — Table S2. Bivariate correlations of study predictor and criterion variables (DOCX 35 kb) [file 13031_2019_201_MOESM1_ESM.docx]

Additional file 1: Table S2 Bivariate correlations of study predictor and criterion variables

|  | |  | **1** | **2** | **3** | **4** | **5** | **6** | **7** | **8** | **9** | **10** | **11** | **12** | **13** | **14** | **15** | **16** | **17** | **18** | **19** | **20** | **21** | **22** | **23** |  |
| --- | --- | --- | --- | --- | --- | --- | --- | --- | --- | --- | --- | --- | --- | --- | --- | --- | --- | --- | --- | --- | --- | --- | --- | --- | --- | --- |
| 1 | **PTGI Total Score** |  | 1.000 |  |  |  |  |  |  |  |  |  |  |  |  |  |  |  |  |  |  |  |  |  |  |  |
|  | **PTGI Subscales** |  |  |  |  |  |  |  |  |  |  |  |  |  |  |  |  |  |  |  |  |  |  |  |  |  |
| **2** | PTGI Factor 1: Relating to others |  | .931^**^ | 1.000 |  |  |  |  |  |  |  |  |  |  |  |  |  |  |  |  |  |  |  |  |  |  |
| **3** | PTGI Factor 2: New Possibilities |  | .935^**^ | .843^**^ | 1.000 |  |  |  |  |  |  |  |  |  |  |  |  |  |  |  |  |  |  |  |  |  |
| **4** | PTGI Factor 3: Personal Strength |  | .921^**^ | .812^**^ | .842^**^ | 1.000 |  |  |  |  |  |  |  |  |  |  |  |  |  |  |  |  |  |  |  |  |
| **5** | PTGI Factor 4: Spiritual Change |  | .732^**^ | .609^**^ | .595^**^ | .644^**^ | 1.000 |  |  |  |  |  |  |  |  |  |  |  |  |  |  |  |  |  |  |  |
| **6** | PTGI Factor 5: Appreciation of Life |  | .830^**^ | .691^**^ | .736^**^ | .705^**^ | .718^**^ | 1.000 |  |  |  |  |  |  |  |  |  |  |  |  |  |  |  |  |  |  |
| **7** | **LOT-R** |  | .286^**^ | .308^**^ | .284^**^ | .237^*^ | 0.122 | 0.149 | 1.000 |  |  |  |  |  |  |  |  |  |  |  |  |  |  |  |  |  |
|  | **COPE Subscales** |  |  |  |  |  |  |  |  |  |  |  |  |  |  |  |  |  |  |  |  |  |  |  |  |  |
| **8** | Positive reinterpretation |  | .336^**^ | .271^**^ | .303^**^ | .356^**^ | .250^*^ | .242^*^ | .285^**^ | 1.000 |  |  |  |  |  |  |  |  |  |  |  |  |  |  |  |  |
| **9** | Active coping |  | 0.141 | 0.069 | 0.128 | 0.122 | 0.170 | 0.071 | 0.154 | .604^**^ | 1.000 |  |  |  |  |  |  |  |  |  |  |  |  |  |  |  |
| **10** | Planning |  | .282^**^ | .246^*^ | .261^**^ | .248^*^ | .203^*^ | .196^*^ | .294^**^ | .729^**^ | .769^**^ | 1.000 |  |  |  |  |  |  |  |  |  |  |  |  |  |  |
| **11** | Acceptance |  | 0.100 | 0.101 | 0.089 | 0.129 | 0.027 | 0.065 | 0.139 | .469^**^ | .320^**^ | .497^**^ | 1.000 |  |  |  |  |  |  |  |  |  |  |  |  |  |
| **12** | Restraint |  | 0.142 | 0.100 | 0.099 | 0.169 | 0.154 | 0.135 | 0.124 | .486^**^ | .491^**^ | .587^**^ | .443^**^ | 1.000 |  |  |  |  |  |  |  |  |  |  |  |  |
| **13** | Suppression of competing activities |  | 0.157 | 0.155 | 0.129 | 0.172 | 0.091 | 0.021 | .196^*^ | .430^**^ | .471^**^ | .581^**^ | .385^**^ | .404^**^ | 1.000 |  |  |  |  |  |  |  |  |  |  |  |
| **14** | Denial |  | -0.005 | 0.068 | 0.060 | -0.023 | -0.054 | -0.054 | 0.101 | 0.105 | 0.094 | 0.148 | .307^**^ | .357^**^ | .269^**^ | 1.000 |  |  |  |  |  |  |  |  |  |  |
| **15** | Behavioural disengagement |  | -.249^*^ | -0.177 | -0.187 | -.277^**^ | -0.192 | -.205^*^ | -.246^*^ | -0.177 | -0.079 | -0.093 | 0.054 | -0.043 | 0.166 | .356^**^ | 1.000 |  |  |  |  |  |  |  |  |  |
| **16** | Mental disengagement |  | 0.141 | .210^*^ | 0.106 | 0.105 | 0.193 | 0.078 | .259^**^ | .443^**^ | .531^**^ | .593^**^ | .475^**^ | .475^**^ | .502^**^ | .369^**^ | 0.147 | 1.000 |  |  |  |  |  |  |  |  |
| **17** | Use of emotional social support |  | 0.010 | 0.052 | -0.043 | -0.056 | -0.063 | -0.002 | 0.066 | .216^*^ | .234^*^ | .301^**^ | 0.178 | 0.126 | .262^**^ | 0.107 | .376^**^ | .375^**^ | 1.000 |  |  |  |  |  |  |  |
| **18** | Use of instrumental social support |  | 0.074 | 0.150 | 0.044 | -0.007 | 0.024 | 0.011 | 0.069 | .382^**^ | .410^**^ | .504^**^ | .253^**^ | .229^*^ | .342^**^ | .196^*^ | .225^*^ | .429^**^ | .719^**^ | 1.000 |  |  |  |  |  |  |
| **19** | Venting of emotions |  | 0.075 | 0.053 | 0.050 | 0.068 | -0.095 | -0.009 | -0.092 | 0.130 | 0.127 | 0.070 | 0.178 | 0.144 | 0.086 | 0.030 | .195^*^ | 0.037 | .445^**^ | .309^**^ | 1.000 |  |  |  |  |  |
| **20** | Humour |  | -0.030 | -0.041 | -0.054 | 0.039 | -0.071 | 0.012 | 0.094 | .400^**^ | 0.141 | .285^**^ | .215^*^ | 0.177 | 0.059 | 0.077 | -0.028 | 0.150 | .243^*^ | .328^**^ | 0.141 | 1.000 |  |  |  |  |
| **21** | Substance use |  | -.211^*^ | -0.184 | -.224^*^ | -.263^**^ | -0.105 | -0.118 | -.227^*^ | -0.064 | 0.133 | 0.058 | 0.144 | 0.094 | 0.187 | 0.116 | .296^**^ | 0.133 | .242^*^ | 0.158 | .221^*^ | -0.144 | 1.000 |  |  |  |
| **22** | Religion |  | 0.001 | 0.020 | -0.019 | 0.000 | 0.075 | 0.010 | -0.037 | 0.083 | .220^*^ | 0.163 | .280^**^ | .233^*^ | .279^**^ | 0.122 | 0.128 | .199^*^ | 0.159 | 0.177 | .291^**^ | -0.037 | .273^**^ | 1.000 |  |  |
| **23** | **HTQ** |  | 0.121 | 0.129 | 0.147 | 0.171 | -0.011 | 0.134 | 0.035 | 0.063 | 0.004 | 0.089 | 0.036 | .195^*^ | -0.020 | .207^*^ | .246^*^ | 0.022 | 0.032 | 0.038 | 0.101 | -0.032 | 0.164 | -0.136 | 1.000 |  |
| *p < .05 **p < .01  PTGI: Posttraumatic growth inventory  LOT-R: Life-Orientation Test Revised - Optimism-pessimism  COPE (Bosnian): Coping Orientations to Problems Experienced Scale  HTQ (Bosnian): PTSD mean score items 1-16 | | | | | | | | | | | | | | | | | | | | | | | | | | |
